# TRIM31 triggers colorectal carcinogenesis and progression by maintaining YBX1 protein stability through ubiquitination modification

**DOI:** 10.1038/s41419-025-07922-4

**Published:** 2025-08-16

**Authors:** Xiaoqing Li, Ying Wu, Jiahao Guo, Peng Huang, Qiuhui Li, Zhongyu Gao, Yanming Hu, Aidi Gao, Ming Sun, Han Min, Jundong Zhou

**Affiliations:** 1 Suzhou Cancer Center Core Laboratory, The Affiliated Suzhou Hospital of Nanjing Medical University, Suzhou Municipal Hospital, Gusu School, Suzhou, China; 2 Oncology Laboratory of Medical Science and Technology Innovation Center, The Affiliated Suzhou Hospital of Nanjing Medical University, Suzhou Municipal Hospital, Gusu School of Nanjing Medical University, Suzhou, China; 3 Department of Gastroenterology, The Affiliated Suzhou Hospital of Nanjing Medical University, Suzhou Municipal Hospital, Gusu School, Suzhou, Jiangsu China; 4 Huzhou Centre Hospital, Affiliated Centre Hospital Huzhou University, Huzhou, China; 5 Department of Computer Science and Engineering, University of California, Davis, CA USA; 6 The First Clinical Medical College of Xuzhou Medical University, Xuzhou, China; 7 Department of Radiation Oncology, The Affiliated Suzhou Hospital of Nanjing Medical University, Suzhou Municipal Hospital, Gusu School, Suzhou, Jiangsu China; 8 Department of Radiation Oncology, the First Affiliated Hospital of Anhui Medical University, Hefei, Anhui China

**Keywords:** Cancer metabolism, Ubiquitins

## Abstract

Colorectal cancer (CRC) is one of the most common gastrointestinal tumors, and one of the leading causes of cancer-related deaths worldwide. However, the molecular mechanisms underlying CRC development and progression have not been fully elucidated until now. Emerging studies have shown that post-translational modifications of proteins, especially ubiquitination modifications, play an important role in tumorigenesis and progression. Here we identified that the E3 ligase TRIM31, a member of the TRIM (Tripartite Motif) family proteins, is highly expressed during colorectal inflammation-cancer transformation and is associated with poor prognosis in CRC patients. Knockdown of TRIM31 expression led to the suppression of CRC cell proliferation and migration in vitro, tumor formation and metastatic ability in vivo. TRIM31 interacts with YBX1 and catalyses the Lys63 (K63) linkage polyubiquitination of Lys81 on YBX1, which ultimately leads to the stabilization of the YBX1 protein. YBX1 further enhances the stabilization of mRNAs for EREG, GAS6, and MAFG through both m^5^C site-dependent and -independent recognition routes. In addition, activation of NF-κB promotes the binding of P65 to the promoter region of TRIM31 to activate the transcription of the TRIM31 gene. Furthermore, TRIM31 facilitates the entry of P65 into the nucleus, which in turn creates a positive feedback pathway that promotes inflammatory-carcinogenic transformation and tumorigenesis of colorectal. Our findings indicate that TRIM31 may be an important factor driving colorectal carcinogenesis, providing a potential target for intervention in CRC targeted therapy.

## Introduction

Colorectal cancer (CRC) is the most common malignant tumor of the gastrointestinal system, with the fourth highest incidence rate and the second highest mortality rate among all tumors [1, 2]. Over the past decade, although systemic treatment with new therapeutic agents, particularly immune checkpoint inhibitors such as PD-1 antibodies, in combination with other therapeutic modalities, as well as the expansion of locoregional surgical regimens, has significantly improved the overall survival of colorectal cancer patients, the five-year overall survival rate remains unsatisfactory [3, 4]. The metastasis of tumor cells is one reason for treatment failure; however, early diagnosis and intervention could help improve the prognosis of colorectal cancer patients. This necessitates identifying key molecules involved in CRC development and progression and elucidating the underlying molecular mechanisms by which they exert their functions, thereby facilitating the development of new therapeutic strategies.

Recently, there has been increasing evidence that aberrant post-translational modifications (PTMs) of proteins are involved in tumorigenesis and progression through various pathways [5–7]. Ubiquitination, as the most common form of PTM, is widely involved in regulating nearly all tumor cell activities, such as tumor immune response regulation, mitochondrial autophagy, DNA damage repair, and protein degradation, by affecting protein stability, activity, subcellular localization, and interaction with other proteins [8, 9]. The TRIM family is one of the E3 ligase families, comprising at least 80 members. TRIM proteins contain three conserved structural domains: an N-terminal Really Interesting New Gene domain (RING domain), one or two B-BOXes (B1/B2) domains, and a coiled coil (CC) domain. Most TRIM proteins have E3 ubiquitin ligase activity, which mediates the ubiquitination of target proteins and plays important roles in cellular processes such as inflammation, innate immunity, antiviral response, and carcinogenesis [10–12]. For instance, TRIM21 binds to residue R95 of the M1 protein and promotes K48 ubiquitination of M1 at K242, leading to proteasome-dependent degradation and further inhibition of the replication of influenza A viruses (IAV), including H3, H5, and H9 [13]. Additionally, TRIM17 interacts with BAX and promotes its ubiquitination and proteasomal degradation, thereby inhibiting BAX-dependent apoptosis in both the absence and presence of apoptotic stimuli in gastric cancer cells [14].

TRIM31 is a member of the TRIM family, which has been found to regulate antifungal immunity by promoting the binding of its plasma membrane translocation to the C-type lectin receptor via K27 polyubiquitination of SYK [15]. Additionally, TRIM31 promotes the proteasomal degradation of MAP3K7 in the TGF-β1 signaling pathway, which plays a key role in hypertensive nephropathy [16]. Moreover, TRIM31 mediates the ubiquitination and degradation of the TSC1-TSC2 complex, leading to the activation of the mTORC1 pathway and progression of hepatocellular carcinoma [17]. Additionally, TRIM31 directly binds to NLRP3 and promotes K48-linked polyubiquitination and subsequent degradation of NLRP3. TRIM31 deficiency enhances NLRP3 inflammasome activation and exacerbates inflammation in a peritonitis model. In contrast, in inflammatory bowel disease models where NLRP3 exerts a protective role, TRIM31 deficiency conversely alleviates disease severity [18]. These findings suggest that TRIM31 is an important regulatory molecule involved in the regulation of the immune response and tumor progression; however, the role and molecular regulatory mechanisms of TRIM31 in colorectal cancer development remain unclear.

YBX1 is a canonical DNA/RNA-binding protein. Previous studies have demonstrated that the target genes regulated by YBX1 are closely associated with tumorigenesis, progression, and metastasis [19]. Recently, YBX1 was identified as a critical reader of m^5^C modifications, promoting RNA stabilization through recognition of m5C modification sites within RNA molecules. For instance, YBX1 stabilizes SMOX mRNA by recognizing the m^5^C modification site in its coding sequence, thereby driving the progression of esophageal squamous cell carcinoma through activation of mTORC1 signaling [20]. Furthermore, our previous study demonstrated that YBX1 enhances the stability of SLC7A11 and G6PD mRNAs by recognizing m^5^C modification sites within their 3′ untranslated regions (UTRs) and cooperating with ELAVL1 [21].

In this study, we analyzed the expression profile of TRIM family proteins in CRC via transcriptomic analysis and identified that TRIM31 is significantly overexpressed in CRC tissues and associated with poor patient prognosis. Next, we explore the molecular mechanisms by which TRIM31 exercises oncogene functions in CRC cells. Our findings indicate that TRIM31 is a potential therapeutic target for CRC.

## Methods and materials

### Patient tissue samples

Colon cancer tissue samples were collected from 96 patients who underwent surgical treatment between 2020 and 2024 at Nanjing Medical University Affiliated Suzhou Hospital. All tissue samples were preserved through paraffin embedding. Informed consent was obtained from each patient prior to sample collection. This study was approved by the Institutional Ethics Committee of Nanjing Medical University (Approval No. KL901200).

### Cell culture

Colon cancer cell lines (HT-29, DLD-1, LOVO, SW480, and HCT116) were obtained from the Shanghai Institute of Cell Research (Shanghai, China) and were authenticated using short tandem repeat (STR) profiling. The cells were cultured under optimized conditions: HT-29 and DLD-1 cells were maintained in RPMI-1640 medium, LOVO cells in F12K medium, and SW480 and HCT116 cells in DMEM, with all media supplied by HyClone (USA). Each medium was supplemented with 10% fetal bovine serum (FBS) (Biological Industries) to support cell growth. Cells were incubated at 37 °C in a humidified atmosphere containing 5% CO_2_. All cell lines were regularly tested for mycoplasma contamination and confirmed to be mycoplasma-negative.

### Transfection and establishment of stable cell lines

TRIM31 knockdown was achieved by infecting cells with lentiviral particles containing either a non-targeting control shRNA (shNC) or a specific shRNA targeting TRIM31. Following infection, cells were selected using 1 µg/mL of puromycin. For SW480 cells, a separate strategy was employed: these cells were transduced with lentiviral particles carrying the full coding sequence (CDS) of TRIM31 mRNA, followed by puromycin selection. The expression levels of TRIM31 in the respective cell lines were confirmed through quantitative reverse transcription PCR (qRT-PCR) and western blot analysis.

### RNA extraction and quantitative reverse transcription PCR (qRT-PCR)

Total RNA was extracted from the cells using the RNA-easy Isolation Reagent (Vazyme, China), following the manufacturer’s protocol. The concentration of RNA was measured with a NanoDrop2000 spectrophotometer (NanoDrop, USA). One microgram of the extracted RNA was reverse transcribed into complementary DNA (cDNA) using the RT SuperMix kit (Vazyme, China). For qRT-PCR analysis, SYBR Green qRT-PCR Master Mix (Vazyme, China) was utilized, with β-actin mRNA expression serving as an internal control. QRT-PCR reactions were conducted on a StepOne Plus Real-Time PCR System (Applied Biosystems, USA). Data analysis was performed using threshold cycle (Ct) values, and relative gene expression levels were calculated using the 2^-ΔΔCt method. The sequences of small interfering RNA (siRNA) and the primers for qRT-PCR are provided in Supplementary Tables 1 and 2, respectively.

### Western blot analysis

Cells were harvested and lysed using RIPA buffer supplemented with protease inhibitors. The lysates were then centrifuged at 12,000 rpm for 15 min at 4 °C to remove cell debris. Protein concentrations were determined, and ~30 μg of total protein per sample of protein were separated by 10% SDS-PAGE. The separated proteins were subsequently transferred onto polyvinylidene difluoride (PVDF) membranes (Millipore, MA). Membranes were blocked with a 5% non-fat milk solution for 1 h at room temperature, followed by overnight incubation at 4 °C with primary antibodies. After primary antibody incubation, membranes were incubated with the appropriate secondary antibodies for 1 h at room temperature. Protein bands were visualized using an enhanced chemiluminescence (ECL) detection system (Beyotime Biotechnology, China). β-actin expression was used as an internal loading control to normalize protein levels across samples. The details of antibodies used are provided in Supplementary Table 3.

### Cell proliferation assay

Cells were seeded into a 96-well plate and transfected as described. Following transfection, cells were incubated for 24, 48, or 72 h. At the designated time points, 10 µL of CCK-8 reagent (Beyotime Biotechnology, China) was added to each well. The plates were subsequently incubated for an additional 2 h at 37 °C. Absorbance was measured at 450 nm using a Thermo Fisher spectrophotometer (USA) to assess cell proliferation.

### Colony formation assay

Approximately 1000 cells were seeded in triplicate into 6-well plates and cultured under standard conditions (37 °C, 5% CO_2_) for 10 days. After the incubation period, cells were fixed with methanol for 10 min and stained with a 0.1% crystal violet solution. Colonies were subsequently counted and photographed to assess colony formation.

### Cell invasion assay

Cell invasion was assessed using Transwell chambers (Corning, USA) pre-coated with Matrigel (Sigma, USA). Cells were seeded into the upper chambers and incubated under hypoxic conditions for 24 h in serum-free medium at 37 °C with 5% CO_2_. The lower chambers were filled with medium containing 20% FBS as a chemoattractant. Following incubation, cells that had invaded through the Matrigel were stained with Wright-Giemsa stain (Nanjing JianCheng Technology, China) for visualization. The number of invaded cells was quantified by counting cells in six randomly selected fields per chamber under a microscope.

### Wound healing assay

Approximately 1 × 10^6^ cells were seeded into each well of a 6-well plate and allowed to attach. A scratch wound was created in the cell monolayer using a sterile pipette tip, and this procedure was repeated three times to ensure consistency across experimental conditions. To assess wound closure, the width of the scratch was measured and imaged microscopically every 24 h. For each condition, three random fields of view were selected, and the migration ratio was calculated using the formula: (initial scratch width − current scratch width)/initial scratch width. The wound areas were quantitatively analyzed using ImageJ 1.8.0 software.

### Immunohistochemical (IHC) analysis

Paraffin-embedded tissue samples were sectioned into 5 µm slices, deparaffinized with xylene, and rehydrated through ethanol gradients. Antigen retrieval was performed in citrate buffer (pH 6.0) for 5 min. Sections were blocked with 5% BSA and incubated overnight at 4 °C with a primary antibody against TRIM31 (Proteintech, UK) at a 1:200 dilution. After incubation with an HRP-conjugated secondary antibody for 1 h, the color reaction was developed using DAB, followed by counterstaining with hematoxylin. The sections were dehydrated and mounted. Immunohistochemical evaluation was performed by two independent pathologists using a Leica microscope (Leica Corporation, Germany). Staining intensity was graded 0–3, and the percentage of positive cells was scored from 0 to 4. The final IHC score was calculated by combining intensity and cell positivity, with expression classified as low (score < 6) or high (score ≥ 6). This study was approved by the Research Ethics Committee of The Affiliated Suzhou Hospital of Nanjing Medical University and all patients who participated offered informed consent.

### Immunoprecipitation and LC-MS/MS analysis

For immunoprecipitation, cells were grown in 10 cm culture dishes, collected, and lysed in RIPA buffer (Beyotime Biotechnology, China) for 15 min at 4 °C. After centrifugation, the supernatant was incubated overnight at 4 °C with the primary antibody (TRIM31, Proteintech, UK) while gently agitating. To capture the antigen-antibody complex, 40 µL/mL of protein A + G agarose beads (Abcam, UK) were added, and the mixture was incubated for an additional 3 h at 4 °C, followed by centrifugation. The immunoprecipitate was washed four times with RIPA buffer and resuspended.

The immunoprecipitated proteins were subjected to liquid chromatography-tandem mass spectrometry (LC-MS/MS) analysis. Peptides were dissolved in 0.1% formic acid (solvent A) and loaded onto a 15 cm, 3/4-inch inner diameter reversed-phase analytical column. The peptides were eluted at a constant flow rate of 400 nL/min using an EASY-nLC 1000 UPLC system. Nanospray ionization (NSI) was employed for ionization, and tandem mass spectrometry was performed on a Q Exactive™ Plus mass spectrometer (Thermo Fisher Scientific). Raw data were processed using Byonic software (Protein Metrics Inc., USA) for protein identification and quantification.

### GST pull-down assay

To examine the interaction between YBX1 and TRIM31, GST pull-down assays were performed. YBX1-GST and TRIM31-His recombinant proteins were expressed in E. coli BL21 (DE3) cells, and purified using glutathione-Sepharose beads and Ni-NTA agarose beads, respectively. For the pull-down assay, 1 µg of YBX1-GST protein was incubated with 20 µL of glutathione-Sepharose beads at 4 °C for 1 h, followed by washing with lysis buffer. The beads were then incubated with 2 µg of TRIM31-His in binding buffer at 4 °C for 3 h. After washing, bound proteins were eluted with SDS sample buffer, and the interaction was analyzed by SDS-PAGE and Western blotting using anti-His and anti-GST antibodies.

### RNA sequencing

The CRC cells were transfected with either siRNA targeting YBX1 (siYBX1) or a control siRNA using Lipofectamine 3000 (Thermo Fisher Scientific) following the manufacturer’s protocol. Each transfection was performed using 50 nM of siRNA in a 6-well plate format. After 48 h, total RNA was extracted using the RNA-easy Isolation Reagent (Vazyme, China). The RNA quality and quantity were assessed using a NanoDrop 2000 spectrophotometer. Only RNA samples with RIN values > 7.0 were used for library preparation, which was performed by BGI Genomics (Shenzhen, China). Sequencing was conducted on an Illumina platform to generate high-quality, paired-end reads. The raw sequencing data were processed, trimmed for quality, and aligned to the human genome (GRCh38) using the STAR aligner. Differential expression analysis was performed using the DESeq2 package in R, considering genes showing a false discovery rate (FDR) < 0.05 and a fold change (FC) ≥ 2 as significantly differentially expressed. Functional enrichment analysis was conducted using gene ontology (GO) and KEGG pathway analyses to elucidate the biological implications of the findings.

### Labeled amino acid-coupled enhanced sequencing (LACE-seq)

Following crosslinking under UV irradiation (600 mJ/cm^2^), the colorectal cancer (CRC) cells were lysed in a lysis buffer composed of 20 mM Tris-HCl (pH 8.0), 1 mM EDTA, 0.1% SDS, 400 mM NaCl, 1 mM DTT, 0.5% Triton X-100, along with protease inhibitors, RNasin Plus, and DNase I. After lysis, RNA was fragmented using 0.1 U/µL MNase, and immunoprecipitation was conducted with 10 µg of YBX1 antibody, utilizing IgG as a control. Following extensive washing, the RNAs were dephosphorylated with FastAP. A linker DNA (AGATCGGAAGAGCACACGTCT) was then ligated to the RNA, and reverse transcription was performed to synthesize complementary DNA (cDNA). The purified cDNA was subsequently processed for sequencing library construction using the KC-Digital™ Stranded RNA Library Prep Kit (Wuhan Seqhealth Co., Ltd.), and the final library was sequenced by Wuhan KangCe Technology Co., Ltd.

### Ubiquitination assay

For the ubiquitination assay, cells were treated with 30 µM MG-132 (TOPSCIENCE, China) for 6 h to inhibit proteasomal degradation. After treatment, cells were lysed, and the lysates were subjected to immunoprecipitation using an anti-YBX1 antibody (Abcam, UK), as described previously. Ubiquitination levels were assessed via Western blotting using an anti-ubiquitin antibody (Santa Cruz Biotechnology, USA).

### RNA stability analysis

CRC cells were cultured to 80% confluence and subsequently transfected with siRNA targeting YBX1 or a non-targeting control siRNA using Lipofectamine 3000 (Thermo Fisher Scientific), at a final concentration of 50 nM per well in a 6-well plate format. Following transfection, cells were treated with 5 µg/mL actinomycin D to inhibit transcription and harvested at designated time points (0, 3, 6, 9, 12, and 24 h post-treatment). Total RNA was extracted using TRIzol reagent (Invitrogen) according to the manufacturer’s instructions. The mRNA levels of EREG, GAS6, and MAFG were quantified using qRT-PCR. GAPDH was employed as a reference gene to normalize the expression levels across different samples. The qRT-PCR reactions were performed in triplicate for each sample to ensure accuracy and reproducibility.

### Methylated RNA immunoprecipitation (MeRIP)

Cells were transfected with siRNA targeting NSUN2 or a negative control siRNA (si-NC) using Lipofectamine 3000 (Thermo Fisher Scientific) at a final concentration of 50 nM in a 6-well plate format. After 48 h, total RNA was extracted using TRIzol reagent (Invitrogen, USA) according to the manufacturer’s protocol. Total RNA was then fragmented using an ultrasonicator to obtain RNA fragments suitable for immunoprecipitation. For the immunoprecipitation step, protein A/G agarose beads were incubated with either an anti-m^5^C antibody or a control IgG antibody at 4 °C for 4 h. Following this, the fragmented RNA was added to the antibody-bound beads in IP buffer (150 mM NaCl, 0.1% NP-40, 10 mM Tris-HCl, pH 7.4, and 0.4 U/µL RNase inhibitor) and incubated for 6 h at 4 °C to allow for the binding of m^5^C-methylated RNA. The immunoprecipitated m5C-modified RNAs were isolated using a magnetic stand and subsequently recovered through proteinase K digestion. RNA was then extracted using TRIzol and subjected to qRT-PCR to quantify the levels of EREG. The enrichment of m^5^C in each sample was calculated by normalizing the qRT-PCR results to the input RNA levels. Statistical analyses were performed to compare the m5C modification levels between the NSUN2 knockdown and control groups, with a significance threshold set at *p* < 0.05.

### Chromatin immunoprecipitation (ChIP) assay

ChIP assays were performed to assess P65 enrichment at the TRIM31 promoter in HT-29 and DLD1 cells. Cells were treated with 1% formaldehyde for 10 min to cross-link proteins to DNA, followed by quenching with glycine. Cell lysates were prepared using a lysis buffer and fragmented by sonication for 5 min. The lysates were then incubated overnight at 4 °C with an anti-P65 antibody. Immunocomplexes were captured using 30 µL of protein A/G Sepharose beads for 2 h at 4 °C. After a series of washes to remove non-specifically bound materials, the complexes were eluted with an elution buffer. To reverse cross-linking, samples were treated overnight at 65 °C, followed by proteinase K digestion at 55 °C. Purified DNA was analyzed using qRT-PCR to quantify P65 enrichment, normalized to input DNA, and compared to IgG controls. Statistical analyses were performed to evaluate differences in P65 enrichment at the TRIM31 promoter.

### Luciferase reporter assay

Promoter sequences for TRIM31 and Phospho-NF-κB p65 were obtained from the UCSC Genome Browser and amplified for cloning. These sequences were then inserted into the PGL4.17 vector. Alongside the negative control vector and the positive control vector (PGL4.51), the constructed PGL4.17 vectors containing the TRIM31 and Phospho-NF-κB p65 promoters were transfected into cells using Lipofectamine 3000 reagent (Invitrogen, USA). A total of 2 μg of each constructed vector was used per transfection in a 6-well plate format. Twenty-four hours post-transfection, cells were harvested, and luciferase activity in the cell extracts was measured using the Dual-Luciferase Reporter Assay System (Promega, USA) and a luminometer (Promega, USA). The relative luciferase activity was calculated by normalizing the firefly luciferase (Luc) signal to the Renilla luciferase (R-Luc) signal.

### In vivo mouse model

Twenty-five female BALB/c nude mice, aged six weeks, were obtained from Shanghai SLAC Laboratory Animal Co. Ltd. (Shanghai, China). The animals were housed in a pathogen-free facility with ad libitum access to standard laboratory chow and distilled water. The mice were randomly assigned to five groups: HT-29 shNC, HT-29 shTRIM31-1, HT-29 shTRIM31-2, SW480 vector, and SW480 TRIM31 overexpression (TRIM31 OE). For tumor implantation, cells were resuspended in phosphate-buffered saline (PBS) at a concentration of 2.0 × 10^6^ cells per milliliter. Each mouse received a subcutaneous injection of 0.10 mL of the cell suspension in the right inguinal area. Tumor dimensions were measured bi-daily using a vernier caliper, and tumor volumes were calculated using the formula: V (mm³) = (length [mm] × width^2^ [mm^2^])/2. After two weeks, the mice were humanely euthanized, and the resulting tumors were harvested for analysis of malignancy markers and histological examination via hematoxylin and eosin (H&E) staining. For the metastatic model, another set of twenty-five female BALB/c nude mice was sourced from the same laboratory. Using identical breeding conditions and grouping as in the subcutaneous model, these mice underwent a tail vein injection of 1.0 × 10^4^ cells. Following a five-week observation period, the mice were humanely euthanized, and lung tissues were harvested for further analysis, which included H&E staining and IHC techniques. All experimental procedures involving nude mice were reviewed and approved by the Animal Ethics Committee at Nanjing Medical University, adhering to the guidelines established by the university’s Animal Care and Use Committee.

### Identification of differentially expressed TRIMs in CRC

All protein coding genes belonging to the tripartite motif containing family were collected from NCBI database and manually double-checked. The top four TRIM clusters were visualized using the karyoplotR [22] package. Afterward, transcriptome profiling CRC datasets that included both tumor and non-tumor samples were collected from GEO. Specifically, two GEO microarray-based datasets (GSE134525 and GSE103512 [23]) were collected for differential expression analyses. GSE134525 included three pairs matched tumor and adjacent tissue samples, and GSE103512 was consisted of 57 tumors and 12 adjacent non-tumor samples. Gene expression and associated clinical metadata were collected via the GEOquery [24] package, optional probe to gene annotation was fetched by utilizing the idmap function of the AnnoProbe package. Differential expression analyses were performed via limma-voom [25] with default parameters. Only probes with FDR (Benjamini & Hochberg) lower than 5% and absolute log_2_ fold change (LFC) higher than log2(0.5) were identified as significant differentially expressed gene probes. For simplicity, genes with multiple significant probes were pruned to keep only the most significant one with highest LFC. Identified significantly activated or repressed TRIMs were visualized via the ComplexHeatmap [26] package. For replication, the aberrant activation of TRIM31 were further confirmed by Wilcoxon test-based comparison of expression of TRIM31 in large (with over 100 patients) CRC cohorts (TCGA-COAD [27], TCGA-READ [27], CPTAC2-Colon [28]) from GDC [29] versus normal colon tissue samples from GTEx [30].

### Survival analysis of TRIM31 in CRC

Normalized gene expression and clinical metadata of three CRC cohorts were collected from GDC (TCGA-COAD), cBioPortal [31] (Rectal_MSK2022 [32]), and GEO (GSE33113 [33]) databases. Survival analysis was performed and visualized via the survminer package. Specifically, the optimal cutpoints for TRIM31 expression were determined via the surv_cutpoint function and log-rank test were employed to examine the significant difference between TRIM31-high and TRIM31-low patient groups.

### Statistical analysis

Statistical analyses were performed using SPSS version 21.0 (IBM, USA), and all graphical representations were generated using GraphPad Prism 8 (GraphPad Software, USA). Results are presented as mean values ± standard error of the mean (SEM) derived from at least three independent experiments. Comparisons between different groups were conducted using Student’s *t* test, while survival data were analyzed using the Kaplan–Meier method. Statistical significance was established at a *P* value of less than 0.05.

## Results

### TRIM31 is highly expressed in CRC and associated with patient’s poor prognosis

**Fig. 1:**
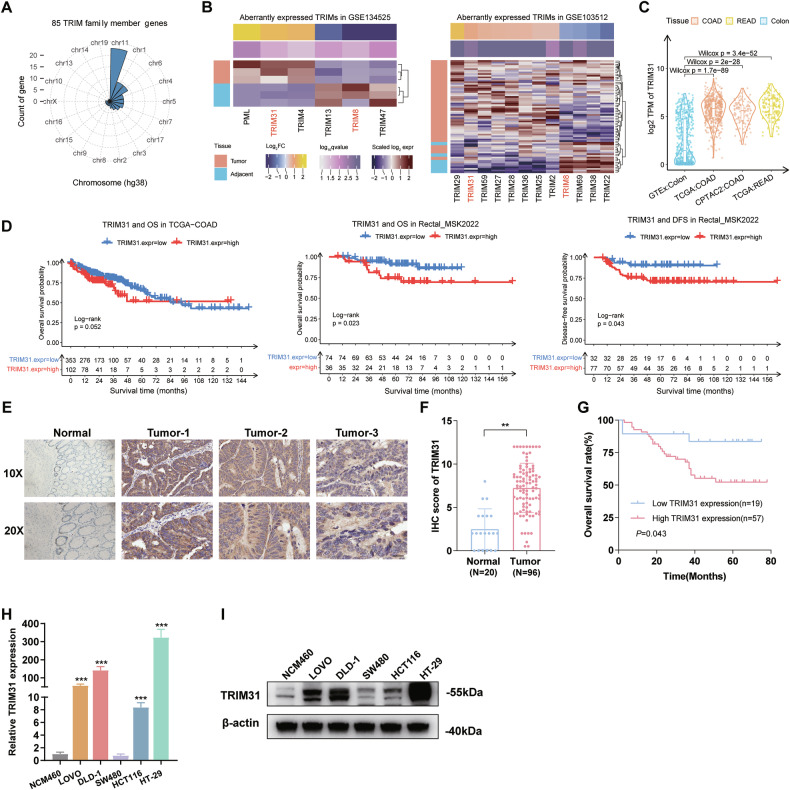
Aberrantly activated expression of TRIM31 and its negative association with prognostic outcome in CRC. **A** The distribution of 85 tripartite motif containing (TRIM) gene family protein-coding genes among all chromosomes. **B** Heatmap analysis of differential expression patterns of the TRIM protein family in colorectal tumor tissue (orange) and adjacent normal tissue (blue) in two independent datasets (GSE134525 [left] and GSE103512 [right]). **C** Box plot depicting TRIM31 expression (log2 TPM values) across four tissue cohorts. **D** Activation of TRIM31 were associated with shorter OS in TCGA-COAD, and worse OS/DFS in Rectal_MSK2022 cohort. **E** Demonstration of TRIM31 immunohistochemical staining differences between normal colon tissue and three colorectal tumors (Tumor-1/2/3) at 10X/20X magnification.Scale bar, 100 µm. **F** The histogram showed that tumor tissue had significantly higher TRIM31 IHC scores than normal tissue, *n* = 24 per group. **G** Survival curves confirmed that patients in the TRIM31 high expression group (*n* = 57) had a lower overall survival rate than those in the low expression group (*n* = 19). TRIM31 expression in normal colorectal cells (NCM460) and colorectal cancer cells, assessed by qRT-PCR (**H**) and Western Blot (**I**), *n* = 3 per group. **P < 0.01; ***p < 0.001.

To explore the expression patterns of 85 TRIM proteins in CRC (Fig. 1A), we downloaded gene microarray data and RNA sequencing data of CRC and normal colorectal tissues from GEO, TCGA, and CPTAC. Differential gene expression analysis showed that TRIM31 was significantly upregulated in CRC tissues compared with normal tissues in both independent datasets, while TRIM8 was down-regulated in both datasets (Fig. 1B). Consistently, TRIM31 expression was also upregulated in CRC tissues from the TCGA and CPTAC datasets (Fig. 1C). Moreover, high TRIM31 expression was associated with shorter overall survival and disease-free survival in CRC patients (Fig. 1D). Next, we confirmed that TRIM31 protein is highly expressed in CRC tissues and has low expression levels in adjacent normal tissues through immunohistochemical staining of our own cohort tissues, and the upregulated TRIM31 expression was significantly associated with shorter overall survival of patients (Fig. 1E–G). Additionally, the results of qRT-PCR and Western blotting showed that the mRNA and protein levels of TRIM31 were remarkably increased in CRC cell lines compared with a normal intestinal epithelial cell line (Fig. 1H, I). These findings indicate that highly expressed TRIM31 may be involved in the development and progression of CRC.

### TRIM31 facilitates CRC cell proliferation and tumor growth in vivo

**Fig. 2:**
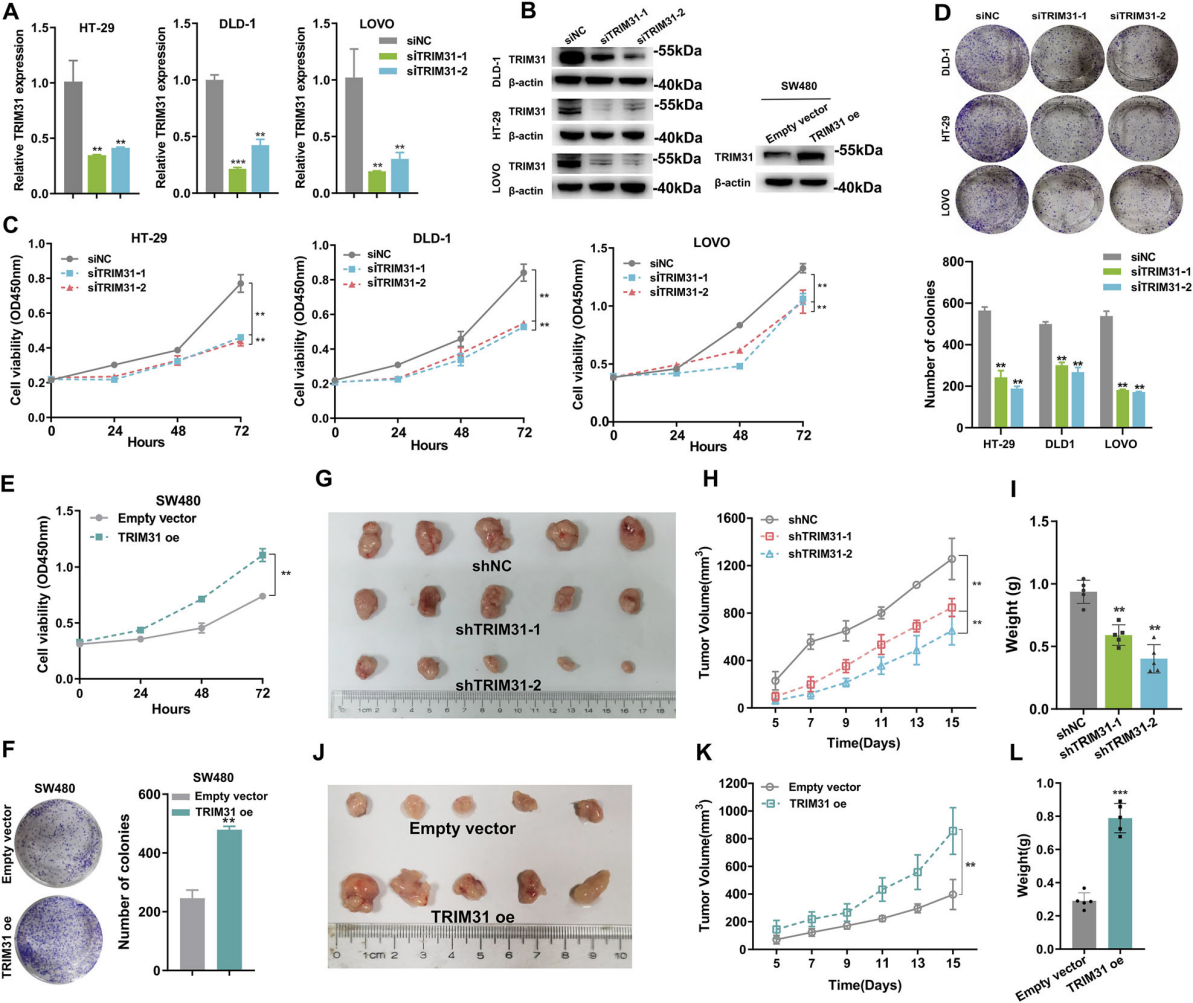
Knockdown of TRIM31 inhibits CRC proliferation in vitro and in vivo. **A** qRT-PCR experiments were performed to detect the efficiency of siRNA to knock down TRIM31 in CRC cells. *n* = 3 per group. **B** Western Blot was performed to detect TRIM31 protein levels after siRNA treatment of DLD-1, HT-29, LOVO cells and after overexpression of TRIM31 in SW480 cells. *n* = 3 per group. **C** Cell viability was determined by CCK-8 assay after knockdown of TRIM31 in HT-29, DLD-1, LOVO cells. *n* = 3 per group. **D** Clone formation assay after knockdown of TRIM31 in HT-29, DLD-1, LOVO cells. *n* = 3 per group. **E** Cell viability was determined by CCK-8 assay after overexpression of TRIM31 in SW480 cells. *n* = 3 per group. **F** Clone formation assay after overexpression of TRIM31 in SW480 cells. *n* = 3 per group. CDX experiments were performed after knockdown of TRIM31 in HT-29 cells, subcutaneous tumors were removed after surgery (**G**) and weighed (**I**), and tumor volumes were measured every 2 days (**H**) *n* = 5 per group. CDX experiments were performed after overexpression of TRIM31 in SW480 cells, subcutaneous tumors were removed after surgery (**J**) and weighed (**L**), and tumor volume was measured every 2 days (**K**) *n* = 5 per group.

To reveal the biological function of TRIM31 in CRC cells, we first knocked down the expression of TRIM31 using siRNA and exogenously upregulated TRIM31 expression levels through lentiviral transduction. The results of qRT-PCR and Western blot confirmed the knockdown and overexpression efficiency (Fig. 2A, B). Next, CCK-8 and colony formation assays showed that knockdown of TRIM31 significantly inhibited the proliferative activity and colony formation ability of CRC cells, whereas overexpression of TRIM31 demonstrated the opposite effect (Fig. 2C–F). Furthermore, results from the mouse xenograft tumor model showed that CRC cell-derived tumors with TRIM31 knockdown grew more slowly and had lighter tumor weights compared to control cells (Fig. 2G–I). In contrast, exogenous overexpression of TRIM31 promoted the growth of xenograft tumors from CRC cells, resulting in heavier tumor weights (Fig. 2J–L). In addition, H&E and IHC staining revealed that the expression level of Ki67 was much lower in tumor tissues derived from CRC cells with TRIM31 knockdown, while the expression level of Ki67 was significantly higher in tumor tissues derived from CRC cells that overexpress TRIM31 (Supplementary Fig. 1A, B).

### TRIM31 promotes CRC cells invasion in vitro and in vivo metastasis

**Fig. 3:**
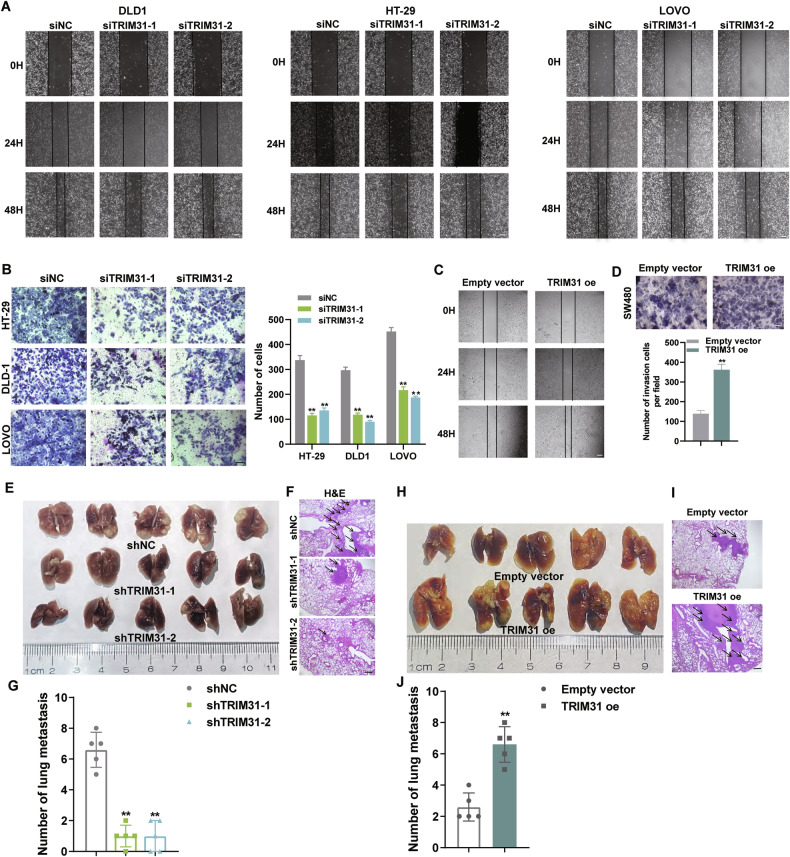
Knockdown of TRIM31 inhibits CRC metastasis in vitro and in vivo. **A** Scratch assay to detect the migration ability of cells after knockdown of TRIM31 in DLD1, HT-29, LOVO cells. *n* = 3 per group. Scale bar, 200 µm. **B** Transwell assay to detect the migration ability of cells after knockdown of TRIM31 in DLD1, HT-29, LOVO cells. *n* = 3 per group. Scale bar, 200 µm. **C** Scratch assay to detect the migration ability of cells after overexpression of TRIM31 in SW480 cells. *n* = 3 per group. **D** Transwell assay to detect cell migration after overexpression of TRIM31 in SW480 cells. *n* = 3 per group. Scale bar, 200 µm. **E** Tail Vein Injection for Lung Metastasis Model to study the metastatic ability of tumor cells in mice after knockdown of TRIM31. *n* = 5 per group. **F** H&E staining of tissues in (**E**). Scale bar, 20 µm. **G** The number of metastatic foci was counted in (**E**). **H** Tail Vein Injection for Lung Metastasis Model to study the metastatic ability of tumor cells in mice after overexpression of TRIM31. *n* = 5 per group. **I** H&E staining of tissues in (**H**). Scale bar, 20 µm. **J** The number of metastatic foci was counted in (**H**). *P < 0.05; **P < 0.01; ***p < 0.001.

Metastasis of tumor cells is one of the main reasons for rapid progression and treatment failure in colorectal cancer. To this end, we further evaluated the effect of TRIM31 expression on the invasion and in vivo metastatic ability of CRC cells. The results of transwell and wound-healing assays showed that down-regulation of TRIM31 significantly inhibited the migratory and invasive abilities of CRC cells (Fig. 3A, B and Supplementary Fig. 1H). In contrast, overexpression of TRIM31 significantly promoted the migratory and invasive potential of CRC cells (Fig. 3C, D and Supplementary Fig. 1I). Moreover, results from the mouse tail vein metastasis model showed that the number of lung metastases was significantly reduced in the mice injected with TRIM31 knockdown CRC cells compared to the control group. However, the number of lung metastatic foci was significantly higher in the group of mice injected with CRC cells exogenously overexpressing TRIM31 compared to the control group (Fig. 3E–J). These findings indicate that TRIM31 may be an important factor driving CRC development and metastasis, but the molecular mechanisms by which it exerts its biological functions remain inadequately defined.

### TRIM31 maintains YBX1 protein stability by catalyzing lysine 81 and 52 sites K63 ubiquitination

**Fig. 4:**
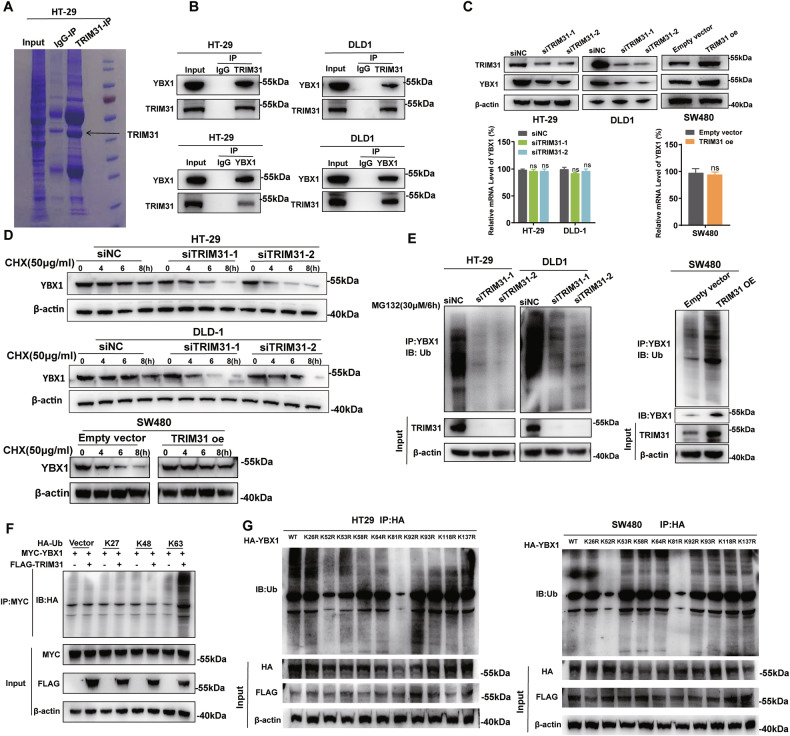
TRIM31 maintains YBX1 protein stability by catalyzing lysine 81 and 52 sites K63 ubiquitination. **A** An Immunoprecipitation in HT-29 cells using TRIM31 antibody followed by Caulmers Brilliant Blue staining. **B** Immunoprecipitation assay to verify the interaction between YBX1 and TRIM31. *n* = 3 per group. **C** Western Blot analysis of changes in YBX1 ‌protein‌ expression levels, ‌combined with qRT-PCR analysis of YBX1 mRNA levels‌, was performed after knockdown or overexpression of TRIM31 in CRC cells. *n* = 3 per group. **D** CHX digestion assay to examine the effect of knockdown or overexpression of TRIM31 on YBX1 stability. *n* = 3 per group. **E** MG132 treatment to examine the effect of knockdown or overexpression of TRIM31 on YBX1 ubiquitination levels. *n* = 3 per group. **F** Immunoprecipitation and Western Blot to detect TRIM31-mediated ubiquitination of YBX1 species. *n* = 3 per group. **G** The ubiquitination level of each lysine of TRIM31 was detected after mutation and overexpression of each lysine to confirm the specific site of ubiquitination of TRIM31. *n* = 3 per group.

To elucidate the molecular mechanism by which TRIM31 exerts its oncogenic function, we performed IP assays using an anti-TRIM31 antibody in HT-29 cells, followed by mass spectrometry to identify TRIM31-interacting proteins (Fig. 4A). The mass spectrometry results revealed that TRIM31 mainly binds to RNA-binding proteins, among which YBX1 attracted particular attention. Subsequent IP assays using lysates from HT-29 and DLD-1 cells confirmed the interaction between TRIM31 and YBX1 (Fig. 4B). Furthermore, knockdown of TRIM31 led to a decreased YBX1 protein level in HT-29 and DLD-1 cells, whereas exogenous expression of TRIM31 increased YBX1 protein levels in SW480 cells, but does not affect the mRNA level of YBX1 (Fig. 4C). In addition, TRIM31 silencing significantly reduced the half-life of YBX1 in HT-29 and DLD-1 cells, while overexpression of TRIM31 enhanced YBX1 protein stability in SW480 cells (Fig. 4D and Supplementary Fig. 1J). These results suggest that TRIM31 stabilizes YBX1 at the protein level. Moreover, TRIM31 knockdown markedly reduced the ubiquitination of YBX1 in HT-29 and DLD-1 cells, while TRIM31 overexpression had the opposite effect (Fig. 4E). The GST pull-down assay further confirmed the interaction between YBX1 and TRIM31 (Supplementary Fig. 1D). Given that TRIM31 can mediate different types of ubiquitin chain linkages, we co-expressed YBX1, TRIM31, and various ubiquitin mutants (K27, K48, and K63) in 293T cells and performed IP assays. The results indicated that TRIM31 specifically catalyzes K63-linked polyubiquitination of YBX1 (Fig. 4F). To determine which lysine residues in YBX1 are targeted by TRIM31 for K63-linked ubiquitination, we generated a series of lysine-to-arginine (K-to-R) mutants of YBX1, each tagged with HA. These mutant constructs were co-transfected with K63-only ubiquitin into HT-29 and SW480 cells, followed by IP using an anti-HA antibody. The results demonstrated that TRIM31 primarily catalyzes K63-linked ubiquitination at lysine residues 81 and 52 of YBX1 (Fig. 4G). Collectively, these findings indicate that TRIM31 promotes YBX1 protein stability by mediating K63-linked ubiquitination at lysine residues 81 and 52.

### TRIM31 exerts oncogenic function partially dependent on YBX1

**Fig. 5:**
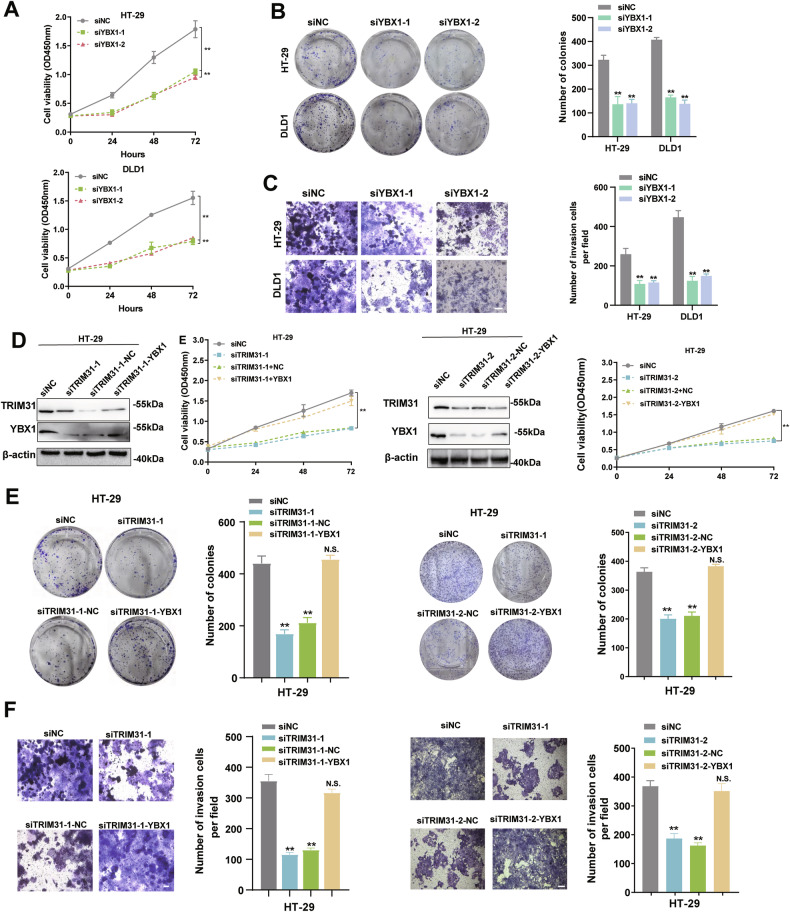
TRIM31 exerts oncogenic function partially through YBX1. **A** Cell viability was determined by CCK-8 assay after knockdown of YBX1 in HT-29 and DLD-1 cells. *n* = 3 per group. **B** Clone formation assay after knockdown of YBX1 in HT-29 and DLD-1 cells. *n* = 3 per group. **C** Transwell assay to detect the migration ability of cells after knockdown of TRIM31 in DLD-1 cells. *n* = 3 per group. Scale bar, 200 µm. **D** Overexpression of YBX1 restored the decreased levels of TRIM31 in HT-29 cells caused by TRIM31 knockdown. *n* = 3 per group. Overexpression of YBX1 restored the reduced proliferation (**E**) and metastatic (**F**) capacity of HT-29 cells caused by TRIM31 knockdown. *n* = 3 per group. Scale bar, 200 µm. ns not significant, *P < 0.05; **P < 0.01; ***P < 0.001.

To determine whether TRIM31 promotes tumorigenesis by regulating YBX1, we first investigated the functional role of YBX1 in CRC cells. CCK-8 and colony formation assays revealed that YBX1 knockdown significantly inhibited cell proliferation and colony-forming ability in CRC cells (Fig. 5A, B), mirroring the inhibitory effects observed upon TRIM31 silencing. Consistently, transwell assays demonstrated that YBX1 knockdown suppressed the invasive capacity of CRC cells to a similar extent as TRIM31 knockdown (Fig. 5C). Importantly, exogenous expression of YBX1 partially rescued the TRIM31 knockdown-induced inhibition of CRC cell proliferation and invasion (Fig. 5D–F). These findings suggest that the oncogenic role of TRIM31 in CRC cells is at least partially dependent on its ability to stabilize and upregulate YBX1.

### YBX1 maintains the mRNA stability of EREG mRNA by recognizing the m5c site

**Fig. 6:**
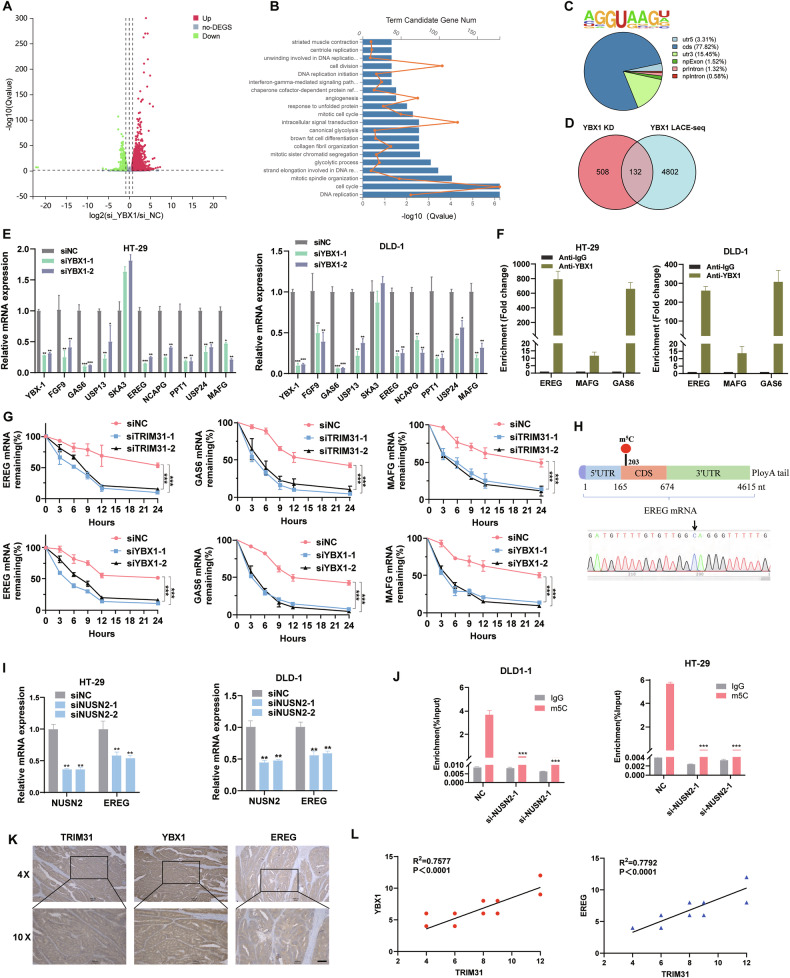
YBX1 enhances the stability of EREG, MAFG, GAS6 mRNA. **A** The volcano plot displays the outcomes of RNA-seq analysis for genes that exhibit differential expression between KO-YBX1 and KO-NC CRC cells. **B** KEGG analysis identifies signaling pathways significantly affected by YBX1 knockdown in CRC cells. **C** Pie chart m5C peaks for percentage of YBX1 high confidence target genes. **D** A total of 132 genes were detected in the intersection of YBX1 post-KD down-regulation and gene fetching detected in YBX1 LACE-seq. **E** Changes in the expression levels of nine genes including FGF9 after knockdown of YBX1 in HT-29 and DLD-1 detected by qRT-PCR. *n* = 3 per group. **F** RIP assay to detect the enrichment of EREG, MAFG, GAS6 RNA on YBX1 protein. *n* = 3 per group. **G** Stability of EREG, MAFG and GAS6 detection after knockdown of YBX1 or TRIM31. *n* = 3 per group. **H** Schematic representation of m5C modification sites and sequencing in EREG mRNAs. Detection of EREG mRNA levels (**I**) and m5C modification levels (**J**) after knockdown of NSUN2. *n* = 3 per group. **K**, **L** IHC staining of TRIM31, YBX1, and EREG in colorectal cancer tissues from 24 patients (**K**). Scale bar, 20 µm. Pearson’s correlation analysis was performed to assess the association between TRIM31 expression and the expression levels of YBX1 and EREG, respectively (**L**). *n* = 24 per group. *P < 0.05; **P < 0.01; ***P < 0.001.

YBX1, an important RNA-binding protein, can regulate RNA stability in the cytoplasm. Our previous studies also showed that YBX1 binds and promotes the stability of mRNAs such as FOXM1 in glioblastoma cells [32]. To clarify the RNA molecules regulated by YBX1 in CRC cells, we performed linear amplification of cDNA ends and sequencing (LACE-seq) to identify YBX1 bound RNAs, and conducted RNASeq analysis in YBX1 knockdown and control cells. The results showed that the expression levels of 640 genes were down-regulated after knockdown of YBX1 in HT29 cells, and these genes were enriched in cell division, intracellular signal transduction and DNA replication etc (Fig. 6A, B). In addition, LACE-seq analysis identified 4934 transcripts bound to YBX1 in HT29 cells. Combined with the RNAseq results, we found that 132 mRNAs both bound directly to YBX1 and their expression levels were down-regulated following knockdown of YBX1 (Fig. 6C, D). Next, top9 genes (FGF9, GAS6, USP13, SKA3, EREG, NCAPG, PPT1, USP24, MAFG) were selected for validation. The results of qRT-PCR showed that the expression levels of all 8 candidate genes, except SKA3, were down-regulated after silencing YBX1. Among these 8 candidate genes, EREG, MAFG and GAS6 were most significantly down-regulated genes (Fig. 6E), and. Subsequently, RIP assay confirmed that YBX1 could bind to the mRNAs of EREG, MAFG and GAS6 in CRC cells (Fig. 6F). Moreover, knockdown of YBX1 and TRIM31 expression resulted in decreased mRNA stability of EREG, MAFG and GAS6 (Fig. 6G). Emerging studies have confirmed that YBX1 can act as an m5C reader and promote mRNA stabilization by recognizing m5C-modified cytosine bases in mRNA sequences. Interestingly, we identified potential m5C modification sites in the mRNA sequence of EREG by bioinformatics analysis. Moreover, the results of RNA-Bisulfite-sequencing confirmed the presence of m5C modification at the C203 site in the mRNA of EREG (Fig. 6H). Furthermore, knockdown of RNA methyltransferase NSUN2 significantly reduced the mRNA expression level of EREG and level of M5C modification in CRC cell(Fig. 6I, J). IHC staining of CRC tissues from 24 patients revealed heterogeneous expression patterns of TRIM31, YBX1, and EREG. Pearson’s correlation analysis demonstrated that both YBX1 and EREG protein expression levels were significantly positively correlated with TRIM31 expression (Fig. 6K, L). These data indicate that YBX1 may promote mRNA stability of EREG by recognizing the m5C site, while mediating mRNA stabilization of MAFG, GAS6 and other target genes in a m5C-independent manner.

### Activated NF-κB promotes the transcription of TRIM31 in CRC cells

**Fig. 7:**
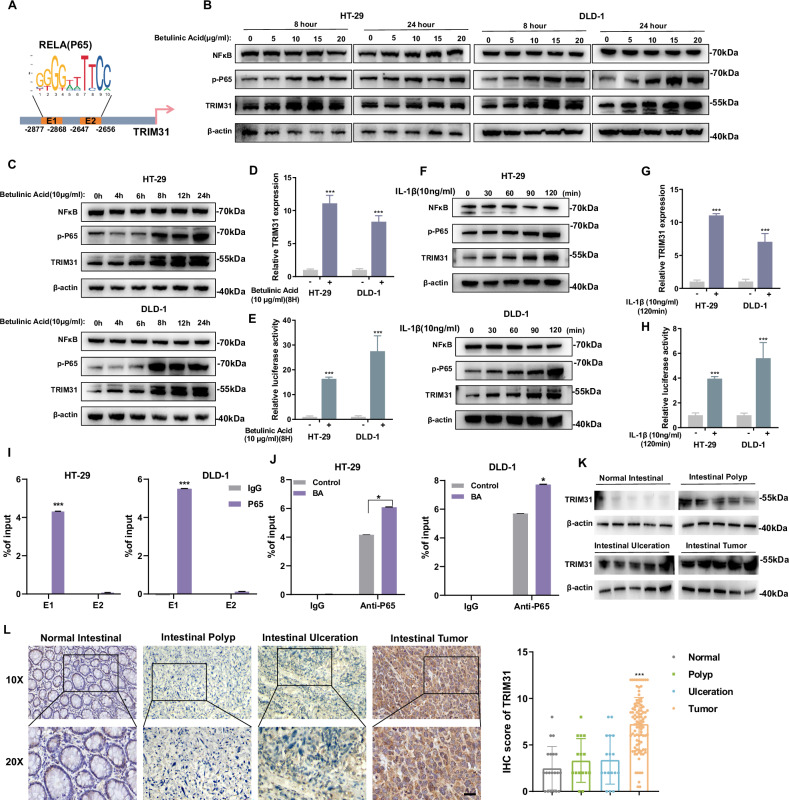
Activated NF-κB promotes the transcription of TRIM31 in CRC cells. **A** Schematic showing potential transcription factor binding sites for TRIM31 analyzed by JASPAR. Protein levels of TRIM31 in HT-29 and DLD-1 cells after Betulinic Acid treatment in relation to Betulinic Acid dose (**B**) and treatment time (**C**). *n* = 3 per group. Changes in TRIM31 mRNA levels (**D**) and promoter activity (**E**) in HT-29 and DLD-1 cells after 8H treatment with Betulinic Acid. *n* = 3 per group. **F** Changes in protein levels of TRIM31 detected after different times of IL-1β (10 ng/mL) treatment. Changes in TRIM31 mRNA levels (**G**) and promoter activity (**H**) of TRIM31 in HT-29 and DLD-1 cells after IL-1β (10 ng/mL) treatment for 8H. *n* = 3 per group. **I** CHIP experiments show major regions of P65 binding to the TRIM31 promoter. **J** CHIP experiments show the effect of Betulinic Acid treatment on the binding of P65 to the TRIM31 promoter. *n* = 3 per group. Western Blot (**K**) and IHC (**L**) analysis of TRIM31 expression levels in normal intestine, intestinal polyps, intestinal ulcers and intestinal tumors. *n* = 3 per group. *P < 0.05; **P < 0.01; ***P < 0.001. Scale bar, 10 µm.

To further investigate the transcription factors (TFs) responsible for TRIM31 upregulation in CRC cells, we analyzed potential TF binding sites in the TRIM31 promoter region using the JASPAR database. Interestingly, two putative P65 (RELA, a core subunit of NF-κB) binding elements were identified within the TRIM31 promoter (Fig. 7A). Treatment of HT-29 and DLD-1 cells with the NF-κB activators Betulinic Acid and IL-1β led to a significant increase in both mRNA and protein levels of TRIM31 (Fig. 7B–G and Supplementary Fig. 1E–G). To further confirm the transcriptional regulation of TRIM31 by NF-κB, we constructed a luciferase reporter plasmid containing the TRIM31 promoter and transfected it into HT-29 and DLD-1 cells. Subsequent treatment with Betulinic Acid and IL-1β markedly enhanced luciferase activity compared with untreated controls (Fig. 7E, H). ChIP assays further demonstrated that P65 predominantly binds to the E1 site, but not the E2 site, within the TRIM31 promoter. Notably, Betulinic Acid treatment enhanced P65 binding to the E1 site (Fig. 7I, J). NF-κB is a well-known transcription factor involved in inflammation, and previous studies have reported that TRIM31 also participates in immune and inflammatory responses. In pancreatic cancer cells, TRIM31 has been shown to upregulate nuclear P65 levels and sustain NF-κB activation by promoting K63-linked polyubiquitination of tumor necrosis factor receptor-associated factor 2 (TRAF2) [34]. Intriguingly, we observed that TRIM31 knockdown significantly reduced nuclear levels of phosphorylated P65 in HT-29 and DLD-1 cells, while TRIM31 overexpression led to increased nuclear accumulation of phosphorylated P65 in SW480 cells(Supplementary Fig. 1C). These findings suggest that NF-κB activation upregulates TRIM31 transcription, and in turn, TRIM31 enhances NF-κB activation via promoting nuclear accumulation of P65, forming a positive feedback loop. Persistent activation of this NF-κB-TRIM31-NF-κB axis may contribute to inflammation-associated tumorigenesis in CRC. To validate this hypothesis, we examined TRIM31 expression in normal colorectal tissues, intestinal polyps, ulcerative lesions, and colorectal tumor tissues. Both Western blotting and immunohistochemistry (IHC) analyses revealed a significant upregulation of TRIM31 in ulcerative and tumor tissues, with the highest expression observed in tumors (Fig. 7K, L). Collectively, these results indicate that the NF-κB-TRIM31-NF-κB positive feedback loop may drive inflammation-to-cancer transformation and promote CRC development and progression.

## Discussion

The Tripartite motif (TRIM) family comprises a class of post-translational regulators with E3 ubiquitin ligase activity, involved in various biological processes associated with tumorigenesis. These include the regulation of cell proliferation, differentiation, metabolism, and apoptosis, primarily through the ubiquitin-proteasome system, as well as via SUMOylation and epigenetic modulation of gene transcription. In this study, we identified TRIM31, a member of the TRIM family, as significantly upregulated in colorectal cancer (CRC) tissues based on transcriptomic analysis and validation in clinical specimens. Moreover, high TRIM31 expression was closely correlated with poor patient prognosis. Previous studies have reported that TRIM31 plays a critical role in regulating inflammatory responses [18] and is implicated in the pathogenesis of several metabolic diseases, including non-alcoholic fatty liver disease [35], hypertensive nephropathy [16], and liver fibrosis [36]. However, in the present study, we demonstrated that TRIM31 knockdown markedly suppressed the proliferative and invasive capabilities of CRC cells in vitro, as well as tumor growth and metastasis in vivo. Collectively, our findings suggest that TRIM31 may serve as a key oncogenic factor driving colorectal cancer initiation and progression. Nevertheless, the precise molecular mechanisms underlying its oncogenic activity remain to be fully elucidated.

As an important E3 ubiquitin ligase, TRIM31 regulates the stability of substrate proteins and exerts its biological functions primarily by mediating diverse ubiquitination modifications. To identify TRIM31-regulated substrates in CRC cells, we performed IP using anti-TRIM31 antibodies followed by mass spectrometry analysis. This approach revealed a number of RNA-binding proteins that interact with TRIM31, among which Y-box binding protein 1 (YBX1) was of particular interest. YBX1, a member of the RNA-binding protein (RBP) family, is a multifunctional protein characterized by a highly conserved cold shock domain (CSD). It preferentially binds to DNA and RNA sequences and functions as either a transcriptional repressor or activator, thereby modulating the expression of numerous genes involved in cell proliferation, DNA damage recognition and repair, and is implicated in tumorigenesis, metastasis, and chemoresistance [19]. For instance, YBX1 has been shown to bind to the 5′-UTR of CCT4 mRNA and promote its translation, thereby facilitating glioblastoma development through mLST8 folding and activation of the mTOR signaling pathway [37]. In CRC cells, we confirmed the physical interaction between TRIM31 and YBX1 by co-immunoprecipitation. Silencing TRIM31 significantly decreased both the expression level and protein stability of YBX1, indicating that TRIM31 positively regulates YBX1 stability. Given that different types of ubiquitination have distinct effects on protein fate, we further investigated the specific modification mediated by TRIM31. Our results demonstrated that TRIM31 predominantly catalyzes K63-linked polyubiquitination of YBX1, rather than K48- or K27-linked ubiquitination, and that this modification occurs primarily at lysine residues 52 and 81. Importantly, the oncogenic role of TRIM31 in CRC cells was found to be at least partially dependent on its regulation of YBX1 stability. These findings collectively suggest that TRIM31 promotes colorectal tumorigenesis and progression mainly by enhancing the stability of the RNA-binding protein YBX1 via K63-linked ubiquitination.

YBX1, an RNA-binding protein, can function as a transcription factor when translocated to the nucleus, where it activates the transcription of downstream target genes. In the cytoplasm, YBX1 regulates RNA metabolism through multiple mechanisms: it can directly bind to RNA molecules to modulate their stability and translation, and it also acts as an m5C ‘reader’, recognizing m5C sites in transcripts to influence RNA stability. For instance, in triple-negative breast cancer cells, YBX1 has been shown to directly activate the transcription of CTPS1 by binding to its promoter region [38]. In ovarian cancer, YBX1 recognizes m5C-modified CHD3 mRNA and maintains its stability by recruiting the RNA-binding protein PABPC1, thereby enhancing homologous recombination (HR) repair and promoting resistance to platinum-induced apoptosis [39]. In the present study, transcriptome sequencing combined with LACE-seq analysis identified several transcripts directly bound and regulated by YBX1, including EREG, MAFG, and GAS6. Follow-up RNA stability assays, RNA immunoprecipitation (RIP), and bisulfite sequencing revealed that EREG mRNA harbors an m5C modification at nucleotide C203, and YBX1 enhances its stability by recognizing this specific site. In contrast, YBX1 was found to stabilize MAFG and GAS6 mRNAs in an m5C-independent manner, suggesting that YBX1 regulates target transcripts via both m5C-dependent and independent pathways. Accumulating evidence has demonstrated the oncogenic roles of EREG [40], MAFG [41] and GAS6 [42] in various cancers. Therefore, our findings indicate that elevated YBX1 protein levels may promote colorectal cancer progression by recognizing and stabilizing oncogenic transcripts such as EREG, MAFG, and GAS6, thereby activating downstream signaling pathways involved in tumorigenesis.

Inflammatory bowel disease (IBD) is a well-established risk factor for the development of CRC. Zhao et al. reported that TRIM31 deficiency alleviates the severity of dextran sodium sulfate (DSS)-induced colitis, suggesting a potential role for TRIM31 in promoting the progression of IBD [18]. In line with this, our study revealed that activation of NF-κB—one of the key inflammatory signaling pathways—enhances the transcription of TRIM31. Notably, TRIM31 in turn facilitates the nuclear translocation of p65 via K63-linked polyubiquitination of TRAF2, thereby further activating NF-κB signaling and forming a positive feedback loop. We speculate that this feedback loop may contribute not only to the persistence of chronic inflammation, but also to the upregulation of oncogenic factors such as EREG, GAS6, and MAFG through the TRIM31-YBX1 axis, ultimately promoting inflammation-driven malignant transformation in the colorectum. Supporting this hypothesis, further analysis demonstrated that TRIM31 expression was low in normal colorectal tissues, but progressively increased in inflammatory tissues, precancerous lesions, and colorectal cancer tissues, with significantly higher expression in the latter two.

**Fig. 8:**
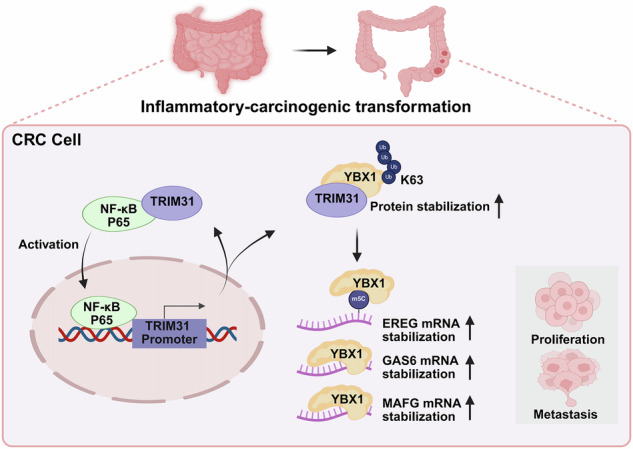
A model diagram to illustrate the findings of this study. TRIM31 directly interacts with YBX1 and catalyzes K63-linked polyubiquitination at YBX1 Lys81, stabilizing the YBX1 protein. Stabilized YBX1 enhances the mRNA stability of oncogenic targets (EREG, GAS6, MAFG) via both m5C-dependent and -independent recognition mechanisms. Furthermore, NF-κB activation promotes P65 binding to the TRIM31 promoter, increasing TRIM31 transcription. TRIM31, in turn, facilitates P65 nuclear translocation, establishing a TRIM31-NF-κB positive feedback loop. This loop perpetuates inflammation, promotes the inflammation-cancer transformation, and fuels colorectal carcinogenesis. TRIM31 thus acts as a central oncogenic driver linking ubiquitination, RNA stabilization, and inflammatory signaling in CRC. This schematic diagram was created using BioRender.com.

In summary, our findings establish TRIM31 as a critical oncogenic E3 ligase that promotes CRC initiation and progression by stabilizing the oncogenic RNA-binding protein YBX1 through K63-linked ubiquitination. Stabilized YBX1, in turn, enhances the mRNA stability of downstream targets including EREG, MAFG, and GAS6 via both m5C-dependent and independent mechanisms. Importantly, we identify a TRIM31-NF-κB positive feedback loop that not only sustains inflammatory signaling but also serves as a molecular bridge linking chronic inflammation to colorectal tumorigenesis (Fig. 8). These findings suggest that TRIM31 may serve as a biomarker for early malignant transformation and represent a potential therapeutic target in CRC.

## Supplementary information

Related Manuscript File

Supplementary Figure-1

western blot raw data

Supplementary Table

## Data Availability

The dataset(s) supporting the findings of this study are included within the article. Requests for materials should be addressed to Ming Sun.
