# Supplementary material for: TRIM31 triggers colorectal carcinogenesis and progression by maintaining YBX1 protein stability through ubiquitination modification
Source: Cell Death Dis. 2025 Aug 16;16(1):621. doi: 10.1038/s41419-025-07922-4 (PMC12357876; doi:10.1038/s41419-025-07922-4)
Supplement: Supplementary file 1 — Related Manuscript File [file 41419_2025_7922_MOESM1_ESM.docx]

**Supplementary Figure 1. Activated NF-κB promotes the transcription of TRIM31 in CRC cells.** **A** **B** Representative images of immunohistochemistry and H&E staining of subcutaneous tumors removed from CDX experiments after knockdown or overexpression of TRIM31. **C** Localization of p-P65 in CRC cells examined after nucleoplasmic separation. **D** GST pull-down assay was performed to verify the interaction between TRIM31 and YBX1 *in vitro*. **E** Quantitative analysis of Western Blot results in Figure 7B. n = 3 per group. **F** Quantitative analysis of Western Blot results in Figure 7C. n = 3 per group. **G** Quantitative analysis of Western Blot results in Figure 7F. n = 3 per group. **H** Quantitative analysis of the scratch assay in Figure 3A. **I** Quantitative analysis of the scratch assay in Figure 3B. Scale bar, 25µm. **J** Quantitative analysis of Western Blot results in Figure 4D. n = 3 per group. *P＜0.05. **P＜0.01.***P＜0.001.
