# Supplementary figures and images for: TRIM31 triggers colorectal carcinogenesis and progression by maintaining YBX1 protein stability through ubiquitination modification
Source: Cell Death Dis. 2025 Aug 16;16(1):621. doi: 10.1038/s41419-025-07922-4 (PMC12357876; doi:10.1038/s41419-025-07922-4)

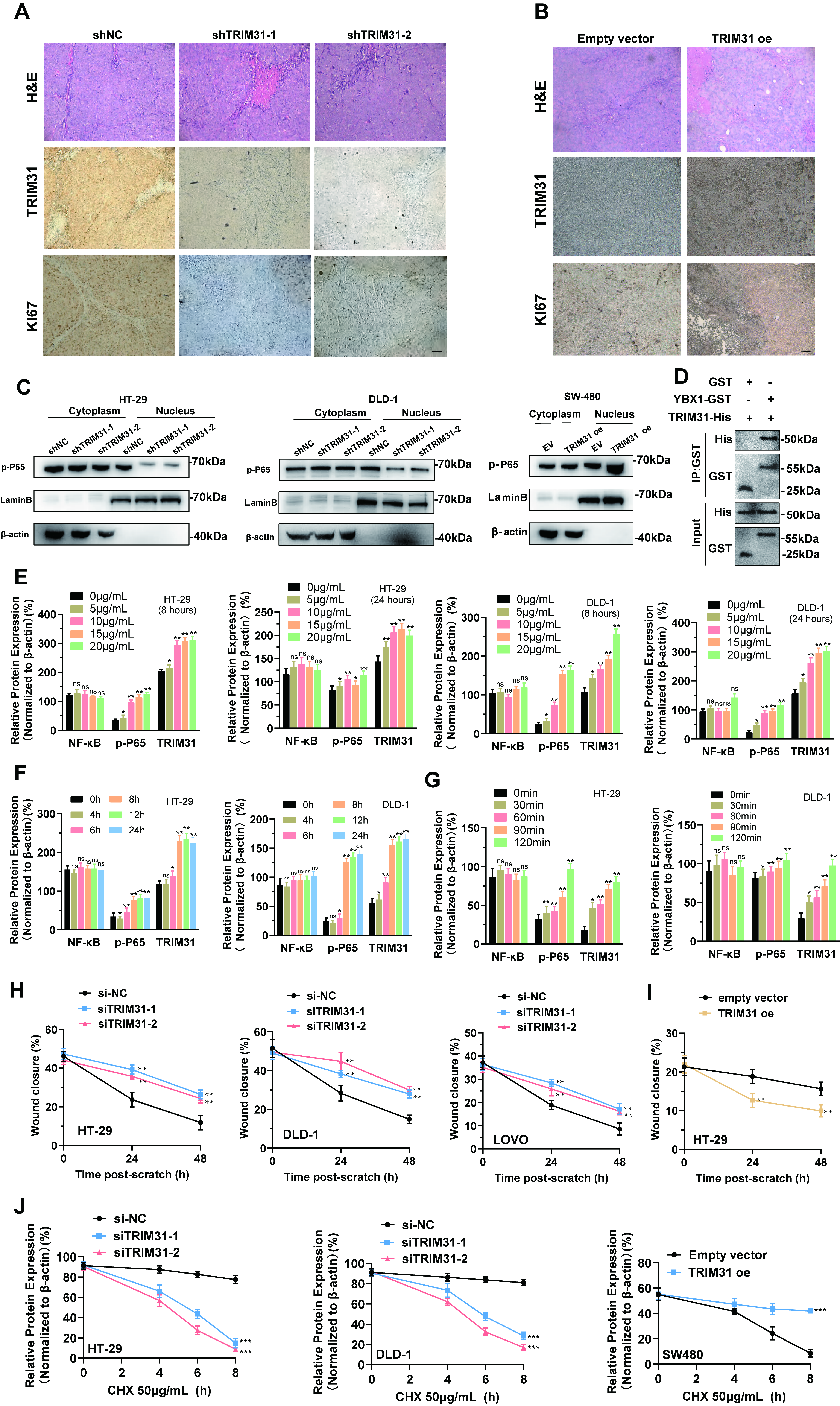

Supplement: Supplementary file 2 — Supplementary Figure-1 [file 41419_2025_7922_MOESM2_ESM.tif]
