# Supplementary material for: TRIM31 triggers colorectal carcinogenesis and progression by maintaining YBX1 protein stability through ubiquitination modification
Source: Cell Death Dis. 2025 Aug 16;16(1):621. doi: 10.1038/s41419-025-07922-4 (PMC12357876; doi:10.1038/s41419-025-07922-4)
Supplement: Supplementary file 4 — Supplementary Table [file 41419_2025_7922_MOESM4_ESM.docx]

**Supplementary Table S1.** Targeted sequences of siRNAs in this study.

| siRNA | Sequences |
| --- | --- |
| si-TRIM31-1  si-TRIM31-2 | GCTCTCAGGATACGAAGACAT  CCTTGTATATTGTAGCTGGAA |
| si-YBX1-1  si-YBX1-2  si-NSUN2-1  si-NSUN2-2 | GGAACGGAUAUGGUUUCAUTT  GCAGACCGUAACCAUUAUATT  GAGATCCTCTTCTATGATCTT  CACGTGTTCACTAAACCCTATTT |

**Supplementary Table S2.** The qRT-PCR primers used in this study

| Gene names | Primers Sequences |
| --- | --- |
| β-actin | Forward: AGCGAGCATCCCCCAAAGTT |
|  | Reverse: GGGCACGAAGGCTCATCATT |
| TRIM31  YBX1  GAS6  USP13  SKA3  FGF9  EREG  NCAPG  PPT1  USP24  MAFG  NSUN2 | Forward: ATGGGGCATTCCTCTGTGTG  Reverse: CATCTAGTCTCTGGCGCAGG  Forward: GGGGACAAGAAGGTCATCGC  Reverse: CGAAGGTACTTCCTGGGGTTA  Forward: TCAACCTGGGAAGTAGAAGTCG  Reverse: GAGTTTCTTCGTGGAGTGATAGTCT  Forward: GGGTCTACAAGAACGAGTGCG  Reverse: CCAGACGCCCCTCTTACCTT  Forward: ATTCCATCTACAAAGAACAGCATA  Reverse: GAAATCGTAGGTGAAGAGGGA  Forward: ACCTGGGTCAGTCCGAAGC  Reverse: GGCTGTGGTCTTTCCTGGTT  Forward: TGCCTGGGTTTCCATCTT  Reverse: GCTACACTTTGTTATTGACACTTGA  Forward: TGCCACCTTGACTACAACTAC  Reverse: TCTGACACCTCCTGTTCGT  Forward: CCCATAAAGGAGGATGTGTA  Reverse: ATTTCACCATCACAAACTTCT  Forward: ATCATTGGTCCATCCCTTAC  Reverse: GGCATACACCTGTCCATAAAC  Forward: GAATGGCACCAGCCTGACGG  Reverse: AGCGTAGCCGCGGTTCTTGA  Forward: GGTATCCTGAAGAACTTGCC  Reverse: ATCTTATGATGAGGCCGCA |

**Supplementary Table S3**. Antibodies used in this study

| Antigens | Manufacturers | Country |
| --- | --- | --- |
| β-actin mAb | AF503,Beyotime Biotechnology | China |
| TRIM31 mAb  YBX1 pAb  UB mAb  HA mAb  MYC mAb  GST mAb  His mAb  EREG mAb  Flag mAb  NFκB pAb  Phospho-NFκB p65  anti-Rabbit  anti-Mouse | 12543-1-AP, Proteintech  ab76149, Abcam  20326, Cell Signaling Technology  3724, Cell Signaling Technology  13987, Cell Signaling Technology  2622, Cell Signaling Technology  2365, Cell Signaling Technology  ab233512, Abcam  14793, Cell Signaling Technology  10745-1-AP, Proteintech  82335-1-RR, Proteintech  A0277, Beyotime Biotechnology  A0286, Beyotime Biotechnology | UK  UK  USA  USA  USA  USA  USA  China  USA  UK  UK  China  China |
